# A case report of primary central nervous system lymphoma with intestinal obstruction as the initial symptom

**DOI:** 10.1097/MD.0000000000010080

**Published:** 2018-03-09

**Authors:** Xiaoke Li, Shuo Qi, Yuntao Jiao, Jing Gao, Hongbo Du

**Affiliations:** a Department of Gastroenterology; b Department of Hematology and Oncology; c Department of Anorectal Surgery, Dongzhimen Hospital, affiliated to BUCM; d Institute of Liver Diseases, BUCM, Beijing, China; e The National Institute of Complementary Medicine, Western Sydney University, Sydney, Australia.

**Keywords:** intestinal obstruction, lymphoma, non-Hodgkin, spinal cord neoplasms

## Abstract

**Rationale:**

Primary central nervous system lymphoma (PCNSL) with initial manifestations of constipation and intestinal obstruction (IO) is rare.

**Patient concerns:**

A 50-year-old Chinese male patient was admitted to the gastroenterology department due to constipation and abdominal distention for 8 days. He had experienced intermittent back pain for 3 years prior to admission. Based on abdominal radiography, he was initially diagnosed with IO and treated with meal restriction and enemas. However, his symptoms worsened, and progressive lower limb weakness was observed.

**Diagnoses:**

A colonoscopy was inconclusive due to the IO. Computed tomography and magnetic resonance imaging revealed space-occupying lesions near centrums 9–11 of the thoracic vertebrae. The patient underwent spinal decompression surgery, and pathologic examination led to a diagnosis of PCNSL (diffuse large B cell lymphoma).

**Outcomes:**

The symptoms of the IO improved postoperatively, and the patient partially recovered his lower limb muscle strength. He returned to his homeland for chemotherapy.

**Lessons:**

IO can be an initial, unspecific symptom of spinal cord compression in patients with PCNSL.

## 1 Introduction

Intestinal obstruction (IO) is a common acute digestive disorder. Common causes include mechanical (tumors, volvulus, or surgical complications), dynamic (paralysis or cramps), and ischemic factors. Primary central nervous system lymphoma (PCNSL) is an extremely rare form of malignant lymphoma that only occurs in <3% of central nervous system (CNS) tumors.^[1]^ The coexistence of these conditions is rare. Herein, we report a case of a male patient who initially demonstrated manifestations of IO with a final diagnosis of PCNSL.

## 2 Case report

A 50-year-old male Chinese patient was admitted due to a lack of defecation for 8 days and with symptoms of abdominal distention. His medical history included a trauma-induced fracture of the 3rd transverse process of the lumbar vertebrae 3 years earlier, after which he intermittently suffered from backache. He received physical therapy (cupping) 3 days prior to admission, which effectively alleviated the pain. His older brother had died from lung cancer. The patient seemed well-nourished. A physical examination was normal; notably, although he reported feeling cold in his lower limbs, the pulse of the dorsalis pedis arteries and sensation in both legs were normal. No enlarged superficial lymph nodes were seen. Percussion revealed a bilateral dullness in the center of his abdomen as well as tympany.

**Figure 1:**
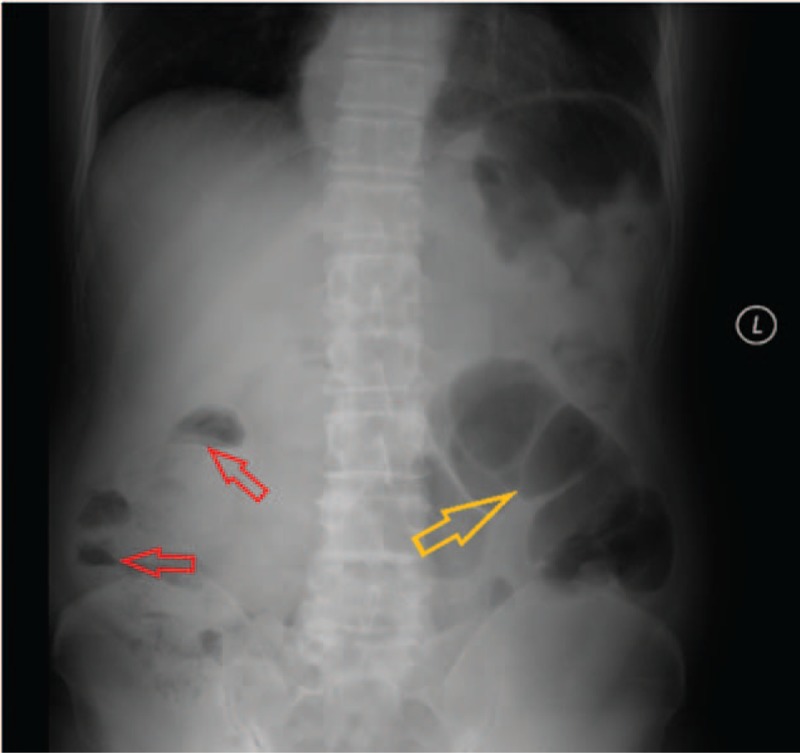
Abdominal radiography revealed air-fluid levels (red arrows) and massive gas accumulation in the dilated intestinal canal (yellow arrow).

Abdominal radiography indicated interspersed gas within the intestinal canal, air-fluid levels (in the right center of the abdomen), and massive intestinal gas accumulation in the dilated intestinal canal (Fig. 1). Laboratory data revealed a slightly elevated concentration of carcinoembryonic antigen, 3.86 ng/mL (reference range, <3.4 ng/mL). Routine blood/urine/stool tests, electrolytes, liver/renal function examinations, human immunodeficiency virus test by ELISA, an upper abdominal ultrasound, and a chest radiography did not show any remarkable findings.

**Figure 2:**
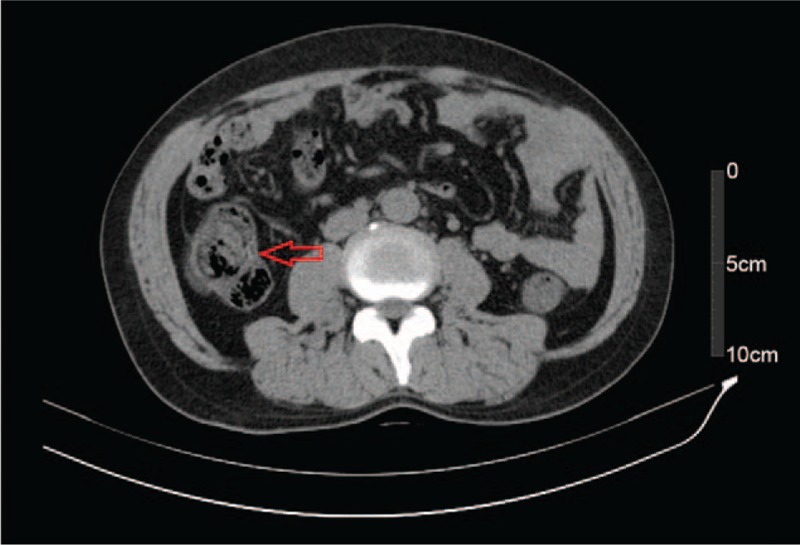
Abdominal computed tomography after admission showed eccentric thickening of the colon walls (red arrow).

**Figure 3:**
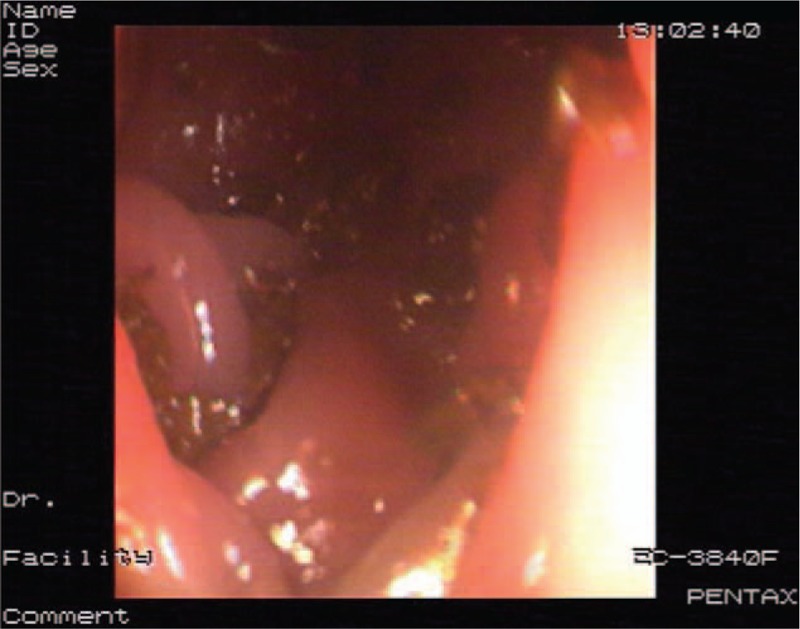
Colonoscopy indicated no obvious space-occupying lesions. An inadequate preparation due to the patient's intestinal obstruction should be noted.

**Figure 4:**
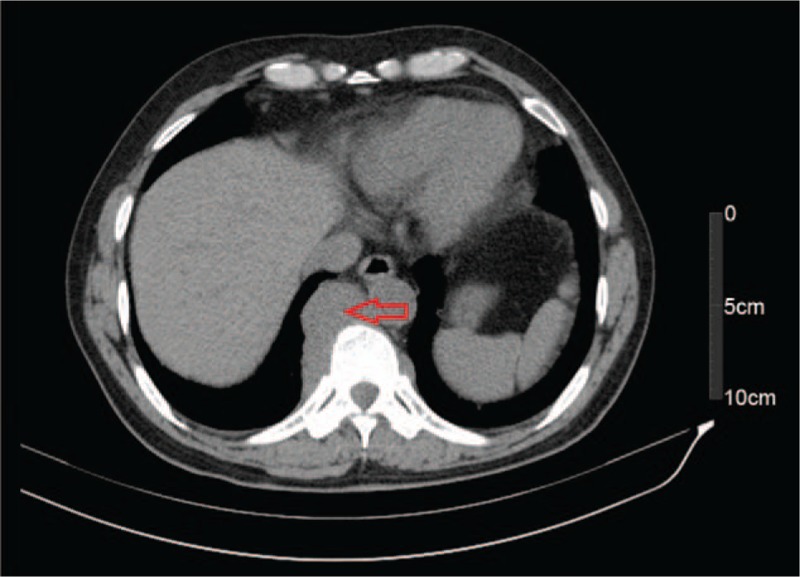
Computed tomography revealed a space-occupying lesion on the right side of centrums 9–11 of the thoracic vertebrae (red arrow).

**Figure 5:**
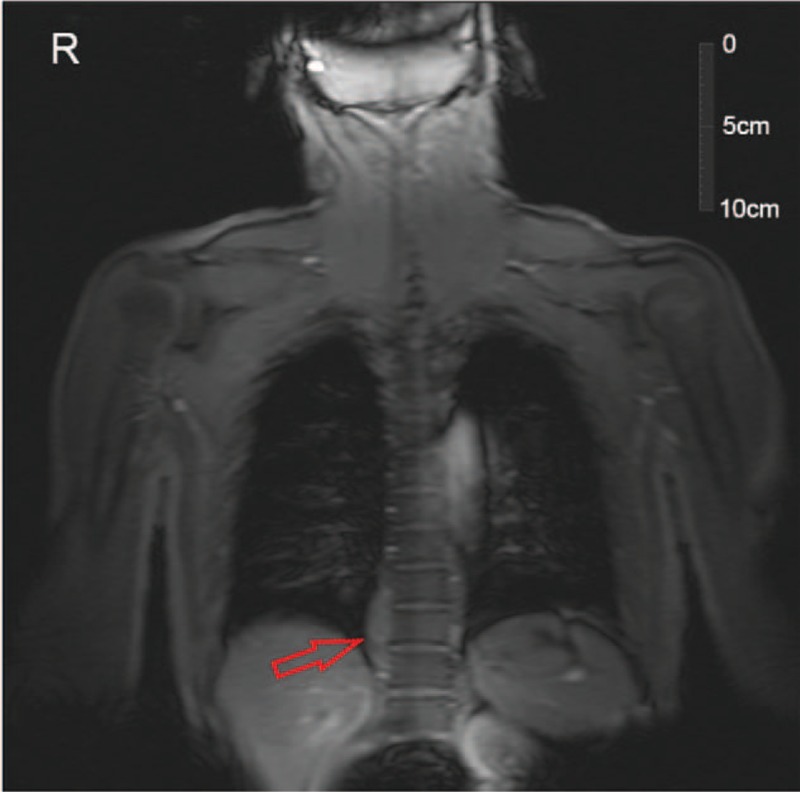
Magnetic resonance imaging (coronal) showed lesions near centrums 9–11 of the thoracic vertebrae (red arrow).

**Figure 6:**
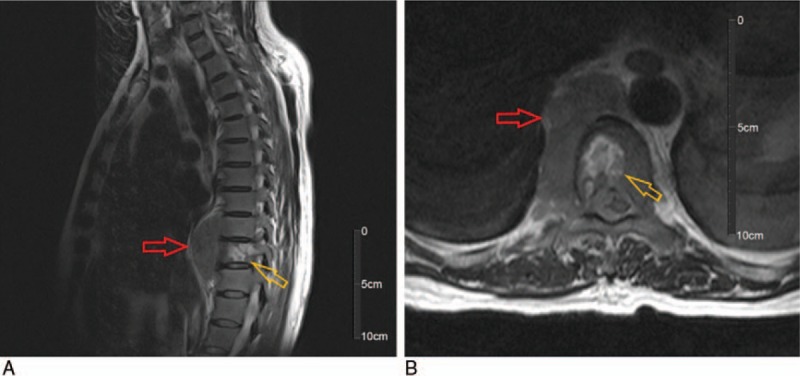
Magnetic resonance imaging (T2-weighted) revealed lesion near centrums 9–11 (red arrow) that invaded the vertebral body (yellow arrow). (A) sagittal and (B) transversal images.

**Figure 7:**
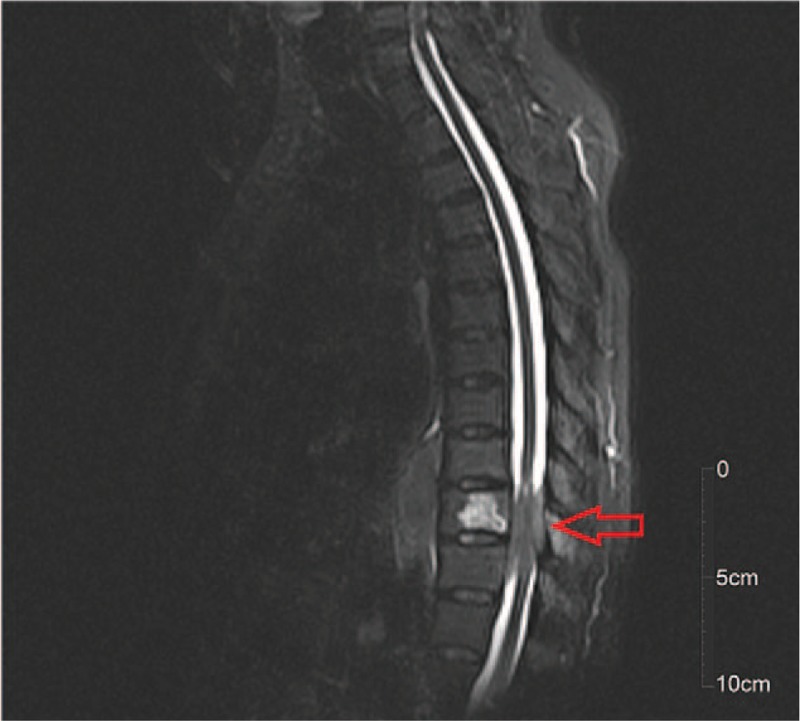
Magnetic resonance imaging (T2-weighted) sagittal images revealed lesions that invaded the spinal canal (red arrow).

Based on these test results, incomplete IO was considered. After admission, the patient continued suffering from abdominal distension as well as a lack of autonomous defecation and flatus although he received conventional therapy, including meal restriction and enemas. Three days after admission, progressive lower limb weakness was observed; the results of the straight leg raise of the left and right legs were 45° (+) and 60° (+), respectively, and that of the reinforced straight leg raise of both legs was (+), with reduction of lower limb muscle strength (left leg: grade II; right leg: grade III) and decreased muscle tension. An axial abdominal computed tomography (CT) scan showed suspicious eccentric thickening of the colon walls (Fig. 2), but no enlarged lymph nodes were seen. No remarkable lesion was found in a subsequent colonoscopy; however, the preoperative preparation was unsatisfactory due to the IO (Fig. 3). Therefore, the CT images were reevaluated with an expanded scope; they revealed a space-occupying lesion to the right of centrums 9–11 of the thoracic vertebrae (Fig. 4). Magnetic resonance imaging of the thoracic spine scan indicated an abnormal signal in centrum 10 of the thoracic vertebrae and space-occupying lesions near centrums 9–11 as well as in the spinal canal (Figs. 5–7). Accordingly, it was assumed that the IO was caused by the space-occupying lesions in the centrums compressing the corresponding spinal cord sections.

**Figure 8:**
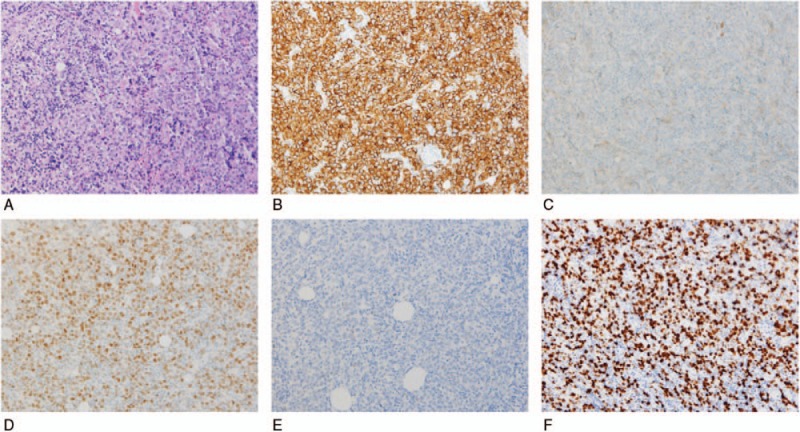
(A) Diffusely distributed atypical (large) lymphoid cells with abundant cytoplasm and visible nucleoli (hematoxylin and eosin staining). (B) Tumor cells showed strong positive staining for the CD20 B cell marker (immunohistochemistry). (C) Tumor cells were negative for CD10 (immunohistochemistry). (D) Over 60% of the tumor cells were positive for Bcl-6 in the nucleolus (immunohistochemistry). (E) Tumor cells were negative for Mum-1 (immunohistochemistry). (F) The Ki-67 index was 40% (immunohistochemistry). Magnification, ×100 for all.

The patient then underwent a resection of the lesions near the centrums. A pathological examination revealed masses in the centrums and right pedicle of the vertebral arch of centrum 10 of the thoracic vertebrae as well as non-Hodgkin lymphoma (NHL) of the diffuse large B cell lymphoma (DLBCL) type (also, germinal-center type). Immunohistochemical analyses showed: AE1/AE3 (−), Bcl-2 (−), Bcl-6 (+), CD10 (−), CD20 (+), CD3 (marginally +), CD30 (Ki-1) (−), CD31 (−), CD34 (−), CD5 (marginally +), HMB45 (−), Ki-67 (index, 40%), Mum-1 (−), and PAX-5 (+) (Fig. 8). The final diagnosis was PCNSL of the DLBCL type, in combination with an IO caused by spinal compression. After the surgery, the IO symptoms were partially relieved, and the patient recovered some of the muscle strength in his lower limbs. He returned to his homeland for chemotherapy and lost to follow-up.

(Important clues and steps for diagnosis are summarized on Table 1.)

**Table 1:**
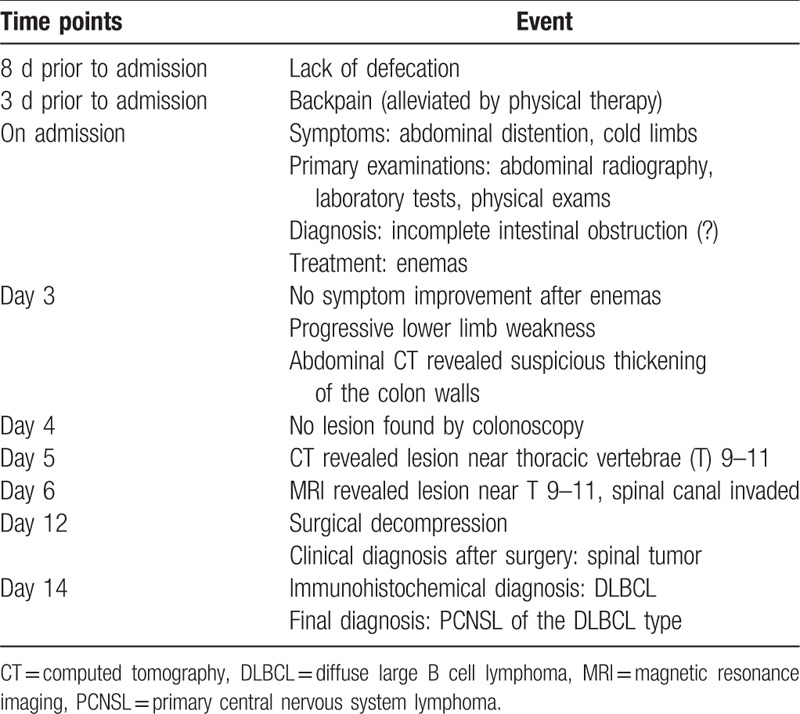
Timeline of the diagnosis and interventions.

## 3 Discussion

Spinal tumors are 10 to 15 times less common than primary intracranial tumors and represent 2% to 4% of all primary tumors of the CNS.^[2]^ They are classified as primary versus secondary tumors, and categorized as intramedullary, intradural, extramedullary, or extradural. The most common invasion sites of spinal tumors include the midthoracic (69%), lumbar (27%), and cervical spine (4%).^[3]^ PCNSL, defined as NHL that can invade not only the brain but also the leptomeninges, eyes, and spinal cord, is extremely rare.^[4]^ Spinal tumors can lead to spinal cord compression and result in a series of unspecific symptoms, such as IO in this case.

PCNSL has specific imaging characteristics. On CT scans, tumor lesions mainly appear as shadows of isodensity or slightly elevated density; on magnetic resonance imaging, they show low or isosignal intensities on T1-WI images and high signal intensities on rho-WI and T2-WI images; gadolinium-diethylenetriamine penta-acetic acid was reported to improve the quality of T1-WI images by markedly enhancing the tumor.^[5]^ Molecular imaging by positron emission tomography with 18F-fluoro-2-deoxy-glucose is helpful for diagnosis, identification of the metabolically active tumor compartment, and prediction of the treatment response.^[6]^

Extranodal NHL accounts for 30% to 40% of all NHL cases in different regions.^[7]^ Spinal tumors are one of the rarest forms of NHL and are only observed in <5% of NHL cases.^[8]^ Leptomeningeal, epidural, and brain metastases are the most common neurologic complications of PCNSL, all of which are associated with a poor prognosis. DLBCL is the most common type of NHL worldwide and the main type of PCNSL.^[9]^

Immunohistochemically, the large lymphoid cells were CD20 (+) and CD3 (−). Spinal DLBCL can be categorized into the germinal center B cell [CD10 (+) or CD10 (−)/BCL-6 (+)/MUM1 (−)] and nongerminal center B cell [CD10 (−)/BCL-6 (−) or CD10 (−)/BCL-6 (+)/MUM1 (−)] types. It is known to initially involve the paraspinal soft tissues, such as the paravertebral ganglion or epidural lymphoid tissue, followed by invasion around the spinal cord via the vertebral foramen without destroying bony structures. This is consistent with the findings in our case.

It has been reported that back pain^[4,10]^ and signs of lower body motor neuron involvement^[11]^ were the most common symptoms in patients with spinal cord compression. In our case, the patient showed similar symptoms. Although the physical examination on admission was normal, he had complained of intermittent back pain for years, which had been assumed to be a sequelae of a past transverse process fracture; however, the abnormal sensation in his lower limbs on admission was unexplained. Unfortunately, we missed the clues until the spinal cord compression syndrome was obvious. In our case, the first symptom that was indicative of a spinal cord compression was the reduction of muscle strength in the lower limbs, which has been reported as a regular manifestation of spinal cord injuries.^[12]^ Due to the high mortality rate of surgery, it was debated whether surgical treatment should be performed.^[13,14]^ The patient survived the surgical decompression, and future chemotherapy as well as radiotherapy is considered beneficial.^[15]^

In our case, the cause of the IO was revealed through surgery and pathological examination. In summary, signs of spinal cord compression, including weakness of the lower limbs and unexplained back pain prior to admission, were observed but inadequate attention was given to these symptoms at the time. Spinal cord injury accompanied by sensory disturbance and dyskinesia of the lower limbs were useful to distinguish this case from other cases of IO. Innervation of the stomach, small intestine, colon, and rectum is mainly formed by the sympathetic nerves of the thoracic (T) 6 to lumbar (L) 3 spinal cord as well as the parasympathetic nerves of the dorsal nucleus of the vagus nerve and sacral parasympathetic nucleus of the sacral (S) 2 to S4 spinal cord. In this case, the spinal cord injury of the thoracic segments impaired both, sympathetic and parasympathetic nerves, and reduced gastrointestinal motility, leading to IO. Moreover, due to the loss of anorectal coordination resulting from forced defecation due to the spinal cord injury, the patient exhibited a lack of autonomous defecation, another reason for his constipation.

The findings of the current case can inspire gastroenterologists when seeking uncommon causes of IO, which is a common disorder encountered in daily clinical practice. A diagnosis of the functional disorder should be based on an understanding of the underlying pathophysiology. PCNSL should be considered as an atypical cause of IO.

## 4 Informed consent

Written informed consent for the publication of this case report and the associated images was obtained from the patient before submission.

## 5 Author contributions

XK.Li was the attending physician of this case and primarily drafted the manuscript; S.Qi and J.Gao were consulting physicians; YT.Jiao was the intern physician, he was involved in the diagnosis and treatment; S.Qi also helped with conducting the literature review; and HB.Du was the chief physician in charge of this case.

## Acknowledgements

The authors thank the patient for his agreement on the publication of this case. The authors also thank professor Lu Zhaohui, vice director of the Pathology Department of the Peking Union Medical College Hospital, for providing the pathology and immunohistochemistry reports as well as the images; Dr Sai Seto of the National Institute of Complementary Medicine, Western Sydney University, Sydney, Australia, for providing language editing and publication support; and the “Outstanding young physician supporting project” (QingMiaoRenCai) of the Dongzhimen Hospital, affiliated to the BUCM, granted to Dr Li Xiaoke for providing the publication fee.

## References

[1] Alvarez-Pinzon AM Wolf AL Swedberg H Primary central nervous system lymphoma (PCNSL): analysis of treatment by gamma knife radiosurgery and chemotherapy in a prospective, observational study. Cureus 2016; 8: e697. 27570717 10.7759/cureus.697 PMC4996544

[2] Chamberlain MC Tredway TL Adult primary intradural spinal cord tumors: a review. Curr Neurol Neurosci Rep 2011;11:320–8. 21327734 10.1007/s11910-011-0190-2

[3] Cho H-J Lee J-B Hur JW A rare case of malignant lymphoma occurred at spinal epidural space: a case report. Korean J Spine 2015;12:177–80. 26512278 10.14245/kjs.2015.12.3.177 PMC4623178

[4] Abrey LE Batchelor TT Ferreri AJ Report of an international workshop to standardize baseline evaluation and response criteria for primary CNS lymphoma. J Clin Oncol 2005;23:5034–43. 15955902 10.1200/JCO.2005.13.524

[5] Nakazawa T Matsuda M Nakasu S Radiological features of primary central nervous system lymphoma. Nihon Geka Hokan 1990;59:141–7. 2130775

[6] Sandu N Popperl G Toubert ME Current molecular imaging of spinal tumors in clinical practice. Mol Med 2011;17:308–16. 21210073 10.2119/molmed.2010.00218 PMC3060992

[7] Li S Young KH Medeiros LJ Diffuse large B-cell lymphoma. Pathology 2018;50:74–87. 29167021 10.1016/j.pathol.2017.09.006

[8] Hirano K Imagama S Sato K Primary spinal cord tumors: review of 678 surgically treated patients in Japan. A multicenter study. Eur Spine J 2012;21:2019–26. 22581192 10.1007/s00586-012-2345-5 PMC3463691

[9] Commins DL Pathology of primary central nervous system lymphoma. Neurosurg Focus 2006;21:E2. 10.3171/foc.2006.21.5.3 17134118

[10] Aabo K Walbom-Jorgensen S Central nervous system complications by malignant lymphomas: radiation schedule and treatment results. Int J Radiat Oncol Biol Phys 1986;12:197–202. 3949570 10.1016/0360-3016(86)90094-5

[11] Flanagan EP O’Neill BP Porter AB Primary intramedullary spinal cord lymphoma. Neurology 2011;77:784–91. 21832220 10.1212/WNL.0b013e31822b00b9

[12] Popescu M Popov V Popescu G Spinal involvement with spinal cord compression syndrome in hematological diseases. Rom J Morphol Embryol 2012;53:1069–72. 23303034

[13] Chang CM Chen HC Yang Y Surgical decompression improves recovery from neurological deficit and may provide a survival benefit in patients with diffuse large B-cell lymphoma-associated spinal cord compression: a case-series study. World J Surg Oncol 2013;11:90. 23601178 10.1186/1477-7819-11-90 PMC3695776

[14] Peng X Wan Y Chen Y Primary non-Hodgkin's lymphoma of the spine with neurologic compression treated by radiotherapy and chemotherapy alone or combined with surgical decompression. Oncol Rep 2009;21:1269–75. 19360303 10.3892/or_00000350

[15] Monnard V Sun A Epelbaum R Primary spinal epidural lymphoma: patients’ profile, outcome, and prognostic factors: a multicenter Rare Cancer Network study. Int J Radiat Oncol Biol Phys 2006;65:817–23. 16542791 10.1016/j.ijrobp.2006.01.002

